# Surface Dosimetry of Patients Undergoing Total Body Irradiation: A Retrospective Analysis for Quality Assurance

**DOI:** 10.7759/cureus.6900

**Published:** 2020-02-06

**Authors:** Arpita Sengupta, Derek R Wilke, Amanda Cherpak, Krista Chytyk-Praznik, Jason Schella, Mammo Yewondwossen, James Allan, Liam Mulroy

**Affiliations:** 1 Radiation Oncology, Nova Scotia Cancer Centre, Queen Elizabeth II Health Sciences Centre, Halifax, CAN; 2 Radiation Oncology, Nova Scotia Health Authority, Dalhousie University, Halifax, CAN; 3 Medical Physics, Nova Scotia Health Authority, Dalhousie University, Halifax, CAN

**Keywords:** clinical dosimetry data, bone marrow transplantation, total body irradiation, quality assurance

## Abstract

Total body irradiation (TBI) is used prior to bone marrow transplantation as part of the conditioning regimen in selected patients. A linear accelerator-based technique was used at our treatment centre between June, 2004 and August, 2015. Patients were treated supine with extended source-to-surface distance (SSD) lateral fields, and prescription dose was 12 Gy delivered in six fractions, two fractions per day. Dose was prescribed to midplane at the level of the umbilicus and monitor units were calculated manually based on measured beam data. Dose variation within 10% of the prescribed midplane dose is considered acceptable for TBI treatment. This was achieved in our clinic by using compensators to account for missing tissue in the head and neck and lower leg regions. Lung attenuators were routinely used to correct for internal inhomogeneity, which resulted from low density lung tissue.

The purpose of this study was to determine whether dose variation was within acceptable limits for these patients as part of a quality assurance process. Following chart review, 129 patients who received six-fraction TBI from 2004 to 2015 were included in this study. Patients receiving single fraction treatment were excluded. Metal oxide semiconductor field effect transistors (MOSFET) dosimetry was used to measure surface dose at four or five locations during patients’ first fraction of TBI. Dosimetry was repeated during the second fraction for any site with variation greater than 10%. Statistical analysis was carried out on patient data, diagnosis and dosimetry measurements. Of the 129 patients who met the inclusion criteria, 50 were diagnosed with acute myelogenous leukemia, 30 with acute lymphoblastic leukemia and 11 with chronic myelogenous leukemia. The rest of the patients were diagnosed with lymphoma or myelodysplastic syndromes. The mean percent variation in dosimetry measurements taken at the specific locations ranged between 3.5% and 8.3%. The highest variation was found in measurements performed on the cheek. A high percentage of all dosimetry readings (85.5%) was within the acceptable range of +10% from the expected value. The highest number of individual readings taken at a specific location that fell outside this range were found at the cheek. We conclude that the linear accelerator delivered TBI at our centre meets the acceptable limits of dose variation over an 11-year period.

## Introduction

Total body irradiation (TBI) is used prior to bone marrow transplantation as part of the conditioning regimen in selected patients. The purpose of TBI is to treat the bone marrow and reduce the number of viable cells and also to suppress the body’s immune system. TBI is complementary to high dose chemotherapy and provides cytotoxicity, immunosuppression and bone marrow ablation [1-3]. The prescription dose point for TBI treatment is typically a point along the midline of the body, at the level of the umbilicus [4]. A 10% dose variation within the entire body, achieved with the use of various beam-modifying devices, is considered acceptable for TBI treatment [1,2,4].

Previous studies have calculated dose delivery using computed tomography-based treatment planning for TBI [5]. Bloemen-Van Gurp et al. carried out in vivo dosimetry using metal oxide semiconductor field effect transistors (MOSFETs) and thermoluminescent dosimeters (TLDs) to verify calculated dose distributions. They compared the treatment planning system (TPS) predicted data with MOSFET and TLD in vivo dose data, and found that mean MOSFET dose values were within 0.5% of TPS data, with a measured dose uncertainty of 3.5% [5]. Patel et al. reviewed a new CT-planned TBI at two institutions and compared it with an older CT-planned TBI technique, demonstrating that in vivo dosimetry measurements are essential in comparing CT-planned data [6,7]. They found that for all three techniques, the in vivo measured doses using diodes were within the acceptable range (+/-10%) of the expected doses.

Quality assurance is an integral part of radiotherapy, ensuring the accurate delivery of doses to patients undergoing radiotherapy [8]. The purpose of our study is to determine that the dose variation measured for TBI patients was within acceptable limits. Adult patients receiving matched unrelated donor transplants (various diagnoses) or those with acute lymphoblastic leukemia (ALL) received TBI as part of the conditioning regimen at the Nova Scotia Cancer Centre (NSCC) between June, 2004 and August, 2015. An extended-SSD (source-to-surface distance), linear accelerator-based technique was used, with dose uniformity was achieved in our clinic with lead compensators to account for missing tissue in the head, neck and lower leg regions. Lung attenuators were also routinely used to correct for dose inhomogeneity due to low density lung tissue. Dose was calculated manually via an in-house spreadsheet, with CT data used only for lung attenuator design. In vivo dosimetry was performed using MOSFETs to measure skin dose at four or five separate locations per patient during the first fraction of TBI. Dosimetry was repeated during the second fraction for any location with variation greater than 10%, or when MOSFET position was noted to have shifted. In the light of these observations, we used a retrospective analysis to explore causes for large dose variations.

## Materials and methods

TBI process at NSCC

Treatment planning for TBI commenced with a conventional CT simulation of patients. A reference point was tattooed at the umbilicus for manual dose calculation. Distances from the reference point to three points superior (head, neck and mid-chest) and three points inferior (mid-thigh, mid-calf and ankle) to the reference point were measured. The separation at these six points and at the reference point were also measured. During the simulation process, a CT image of the thorax region was acquired for designing lung attenuators and calculating thickness of the attenuators. The dose prescription was 1200 cGy to be delivered in six fractions, twice daily with a six-hour minimum interval between fractions. The prescription dose, simulation separation and distance measurements and lung thickness determined from the CT scan were entered into an in-house developed spreadsheet to calculate the monitor units needed to deliver the prescription dose to midplane. The same spreadsheet was used to design the body compensators required to achieve the uniform midplane dose and limit the lung dose to the prescription dose.

Patients were treated in supine position with lateral parallel opposed beams using 18 MV photons. A beam spoiler was used to increase the surface dose. An extended SSD of 500 cm was used to allow treatment of the full body in a single field. In vivo dosimetry using MOSFETs was performed on day 1 with the first fraction. Standard sensitivity MOSFETs from Best Medical (Ottawa, Canada) were used. MOSFETs were taped to the skin surface at specified sites on each patient and delivered doses were read after the first treatment fraction. Measurements were repeated for the following fraction/s if the detector was noted to have fallen off or if the dose variation was greater than 10% from the prescribed midplane dose. Since the beam spoiler increased the surface dose to more than 97% of the midplane dose no additional correction was needed to the MOSFETs reading.

Ethics review, patient selection and dosimetry form review

This project underwent review by the Ethics Review Committee, Nova Scotia Health Authority (NSHA) and received approval. The next step was to collect dosimetry data from the dosimetry reports stored at the NSCC. These dosimetry reports were created as paper documents for each patient receiving TBI during this 11-year period of time. These reports indicate the dose measurements at each designated site on the patient’s body as measured by MOSFETs, the percent deviation from the target dose of 200 cGy as well as set up details including energy, gantry angle, SSD, MU and field size. A brief demographic on each patient is also included in the reports. A review of the dosimetry reports of all patients undergoing TBI from June 2004 to August 2015 was carried out. Patients who underwent TBI in six fractions were included in the study. To facilitate analyzing trends of dose variation between fractions during TBI treatment, patients who underwent TBI in a single dose fraction were excluded from the study. The dosimetry reports from the selected patients were analyzed.

The following data was obtained from the dosimetry reports: patient demographic data (age, gender), diagnosis, MOSFET dosimetry at four sites (cheek, chest, abdomen, leg). Over the 11-year period the MOSFET sites in the lower part of the body had been different and were mostly either upper thigh or ankle with a few in the hip. These data were compiled together under the heading “leg” for consistency and to facilitate reporting of data.

## Results

Demographics

**Figure 1:**
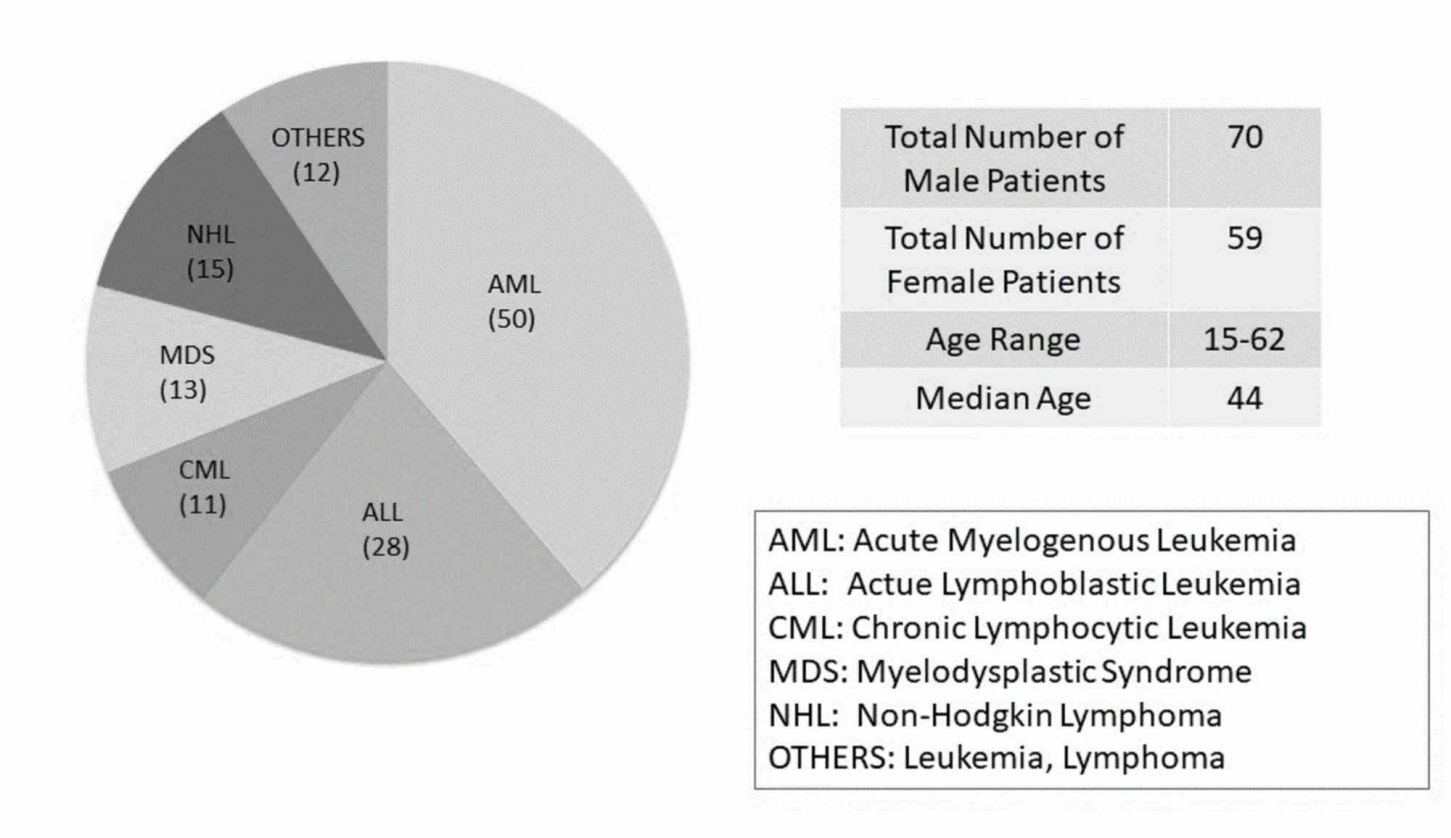
Demographics of patients Demographics of patients included in the study. The figure shows breakdown of patient sex and diagnosis, as well as age range and median.

A total of 129 patients received total body irradiation using a six-fraction regimen between June, 2004 and August, 2015 at the Nova Scotia Cancer Centre. A description of the demographics of the patients including diagnosis is provided (Figure 1).

Of the patients studied, 70 were males and 59 were females (54% and 46%, respectively). Acute myelogenous leukemia (AML) was the most common diagnosis with 50 patients having this condition (39%). There were a total of 28 patients with the diagnosis of acute lymphoblastic leukemia (ALL) (22%). Non-Hodgkin's lymphoma, myelodysplastic syndrome (MDS) and chronic lymphocytic leukemia (CML) constituted 15, 13 and 11 patients, respectively (12%, 10% and 9%). The remaining 12 patients were diagnosed with other types of leukemia and lymphoma (8%) (Figure 1). The above data provides a general idea of the patient population seen at the NSCC and those who are candidates for TBI prior to stem cell transplantation. All these patients were treated with a standard prescription of six fractions of radiation given over three days to a total dose of 1200 cGy [1, 3].

Analysis of dosimetry measurements

**Figure 2:**
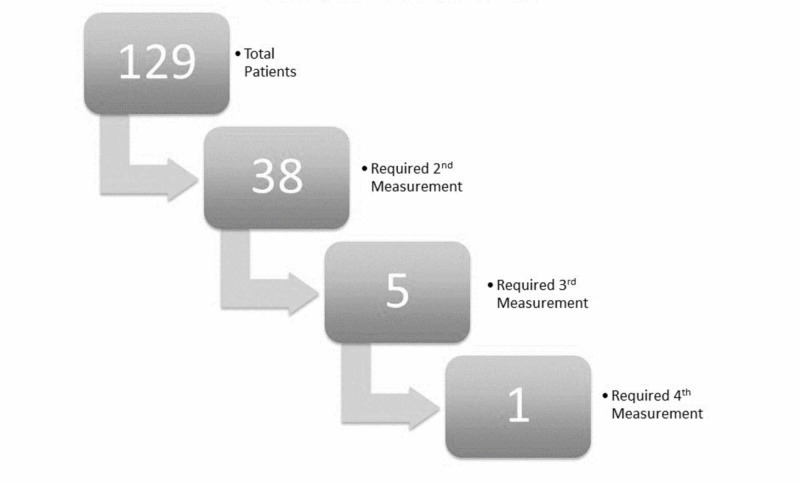
Patients requiring repeat dosimetry measurements Number of patients studied versus number who required repeat measurements due to out-of-range dosimetry. Out of 129 patients, 38 required a second measurement. Out of those 38, five required a third measurement. Out of those five, only one required a fourth measurement.

As per established protocol, dosimetry measurements were repeated for any site during the second fraction when variation was greater than 10%, or when MOSFET position was noted to have shifted. This was done to ensure patients were treated within the acceptable 10% dose variation from the prescribed midplane dose for the remaining fractions of treatment. Out of 129 patients, 38 patients required second measurements, five patients required third measurements and only a single patient required a fourth measurement (Figure 2).

Further analysis of the single patient requiring a fourth measurement is described separately in the discussion section.

Table 1 describes the total number of measurements (including repeat measurements) at each location.

**Table 1:** Mean dosimetry variation at each location Mean dosimetry variation at each location, including all measurements (1st, 2nd, 3rd, and 4th). Variation is shown as an absolute difference from 200 cGy, and is expressed as a percentage of 200 cGy. Standard deviations and medians were calculated likewise.

Location	Cheek/Head	Chest	Abdomen	Leg
Sample size (no. of measurements)	170	163	137	232
Mean % abs. variation (from 200 cGy)	8.4	6.9	5.1	5.7
Std. Dev.	0.1	0.1	0.1	0.1
Median % variation	5.1	5.0	3.8	4.2

**Figure 3:**
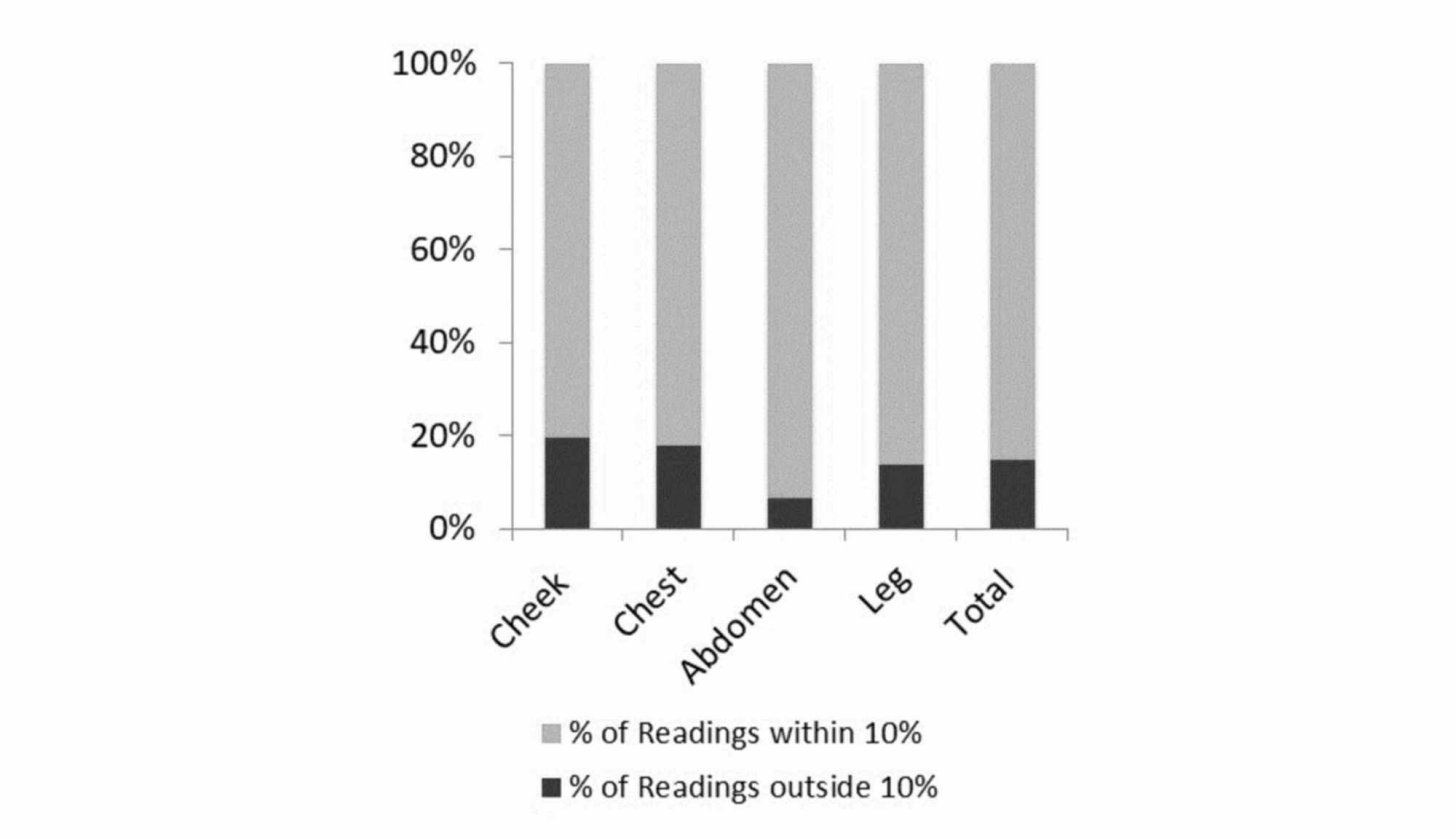
Dosimetry measurements within acceptable range Percentage of dosimetry measurements at each region within and outside acceptable limits. Dosage of 200 cGy ± 10% is considered acceptable.

The mean, median and standard deviation of percent variation of dosimetry measurements at each location are shown. The greatest variation is noted at the cheek. The p-values at all locations are greater than 0.05 indicating that there is no significant difference between measurements. The percentage of readings at all sites (including repeat measurements) that were within plus or minus 10% was 85.3%. The greatest number of readings within the acceptable range occurred at the abdomen, followed by the leg, chest and cheek. These are shown in Figure 3.

Comparison between first measurements and second measurements of 38 patients who underwent second measurements was carried out using a paired T-test. Five of these 38 had a third measurement and one of those five had a fourth measurement which are not included in the paired T-test. The results indicate that there was no significant difference for any dosimetry site (p > 0.05). Therefore, the values between the first and second measurements were statistically similar (Table 2).

**Table 2:** P-values for paired t-test between 1st and 2nd measurements P-values for paired t-test between 1st and 2nd measurements. Note that the percent variations were not converted to absolute values for this calculation.

Paired t-Test 1^st^ Measurements vs. 2^nd^ Measurements				
Location	Cheek	Chest	Abdomen	Leg
P-value	0.29	0.22	0.74	0.17

A five-number summary was computed at each location to determine the spread of data around the median. Interquartile ranges (IQR) for the absolute percent variation at the cheek, chest, abdomen and leg were 6.1%, 5.5%, 4.4% and 5.5%, respectively. This indicates that the data were closely clustered around median measurements. All Q3 values were 8.9% or below, indicating that over three quarters of measurements were within the acceptable ±10% range.

## Discussion

A retrospective analysis of dosimetry measurements for 129 patients was carried out for the period June, 2004 to August, 2015. Most patients did not require a repeat measurement as the first value was within the acceptable plus or minus 10% of the prescribed midplane dose. The analysis shows acceptable variation in dosimetry within 10%, which is indicated by the high percentage of readings (85.9%) within this range.

The second measurements were carried out for patients in which the first measurement was outside the acceptable range of plus or minus 10% of the prescribed midplane dose. This was to determine if there were outliers due to differences in positioning as patients were not rigidly immobilized in this set up. There is also a possibility of inherent uncertainties in MOSFET measurements as well as noted problems in experimental setup such as MOSFETs becoming mispositioned throughout treatment. This was performed as part of quality assurance measure for our centre. However, the paired T-test analysis showed that in all locations, second measurements did not differ significantly from the first as a group. This is contradictory to the fact that measured skin dose tended to be within the acceptable range for the second measurement. It is evident that for patients requiring repeat measurements, the dosimetry values did change on the second measurement. This discrepancy may have resulted because the sample size is not big enough to reflect the change statistically.

This raises the question whether it was worthwhile repeating the measurements. In order to make sure that the dose delivered was within the acceptable range of 10%, it was necessary to obtain measurements at least for the first fraction. This is extremely important for quality assurance purposes. However, our data shows that in cases where the first dose was outside acceptable limits, the second measurement often revealed an acceptable value. Thus, repeating the measurement did have value and allowed for a second chance to evaluate the skin dose. The adjustments made between fractions one and two included verification of patient positioning, proper placement of MOSFET leads and making sure data input to the linac matched the intended dose parameters for the patient. However, in rare cases where measurement had to be repeated beyond fraction two, careful quality assurance was performed for every step. This included physicist checking the plan, making sure all calculations were correct including the energy of the photon beam. These results provide additional assurance that the treatment delivered at this institution is consistent with the current standard [1-4]. Studies have shown the importance of in vivo dosimetry even in the context of CT-based treatment planning using commercial TPS [5,6]. Careful analysis may further identify systematic errors as described in the case below.

There was still a certain percentage of readings that were outside the 10% acceptable range (14.7%). The greatest variation was noticed in the readings for the cheek. This variation could be attributed to various causes. Imperfect compensation, imperfect immobilization, and limitations in lead placement are some of the possible causes. Physical factors for variation in readings also include the lead falling off, detector movement, or patient movement during treatment: this may or may not have been documented consistently. The importance of repeating measurements is evident from the incident described below. In this particular case, the first three measurements were outside the acceptable range and a fourth measurement was necessary. Several factors including human error were responsible for the discrepancies.

One out of 129 patients required a fourth measurement due to dosimetry values at all sites outside the acceptable 10% range. It was discovered after the third fraction, that the energy of the photon beam used was 6 MV instead of the 18 MV which was used for treatment planning. There were several factors that led to this incident. On that particular day, the linear accelerator was having an issue with low output in the range of 3% which made it difficult to interpret the lower dosimetry values read by MOSFETs. The TPS has a default of 6 MV which needed to be changed to 18 MV for TBI delivery. This was missed for this particular patient during the different quality assurance steps. As mentioned earlier, MUs, energy and field parameters are all entered manually into the TPS; this increases the possibility of human error. The treatment being delivered using the wrong energy was discovered after the third fraction due to repeated low values of MOSFET readings. The plan was revised by a physicist using 18 MV photon energy. Dose discrepancy at midline was calculated as -1.3% per fraction, total of 0.7% for the entire treatment. Doses were within the acceptable range of 10% in the fourth fraction, once the energy was corrected and the plan was revised. The patient was treated with the revised plan for the remaining fractions. This is an example of human error which has been detected using in vivo dosimetry. The patient was informed of the incident; successful engraftment of the transplanted marrow did occur and standard follow-up was carried out.

At our centre, we have used MOSFETs for in vivo dosimetry. Characteristics that make MOSFETs attractive for in vivo dosimetry are their small size of dosimetric volume, instant readout, ability to store the accumulated dose and minimal effects of temperature [9,10]. MOSFETs have limitations as well. Accuracy and limited life span of a MOSFET may lead to failure to read. The uncertainty associated with MOSFETs in terms of dose reproducibility is about 3% [9]. The characteristics of MOSFETs in in vivo dosimetry have been studied in detail by Ramaseshan et al. and the accuracy has been shown to be within +5% in terms of verification of dose delivery to patients [11]. This shows that in our study, very few patients had dosimetry measurements outside the acceptable range with MOSFET uncertainty taken into account.

## Conclusions

This retrospective analysis of 129 patients who received TBI during the period June, 2004 and August, 2015 at the NSCC was performed to determine the accuracy of dose delivery using a linear accelerator-based technique. The data shows acceptable variation in dosimetry within 10% for most of the measurements. We conclude that the linear accelerator-delivered TBI at our centre meets the acceptable limits of dose variation, based on results achieved with 129 patients over an 11-year period. This data provides useful quality assurance for the Radiation Oncology Department. This article highlights the utility of quality assurance: careful analysis may lead to the identification and timely correction of systematic errors that may impact patient treatment.
